# Abundance, not diversity, of host beetle communities determines abundance and diversity of parasitoids in deadwood

**DOI:** 10.1002/ece3.7535

**Published:** 2021-04-08

**Authors:** Sebastian Vogel, Andreas Prinzing, Heinz Bußler, Jörg Müller, Stefan Schmidt, Simon Thorn

**Affiliations:** ^1^ Department of Animal Ecology and Tropical Biology (Zoology III) Field Station Fabrikschleichach Julius Maximilians University Würzburg Rauhenebrach Germany; ^2^ Research Unit “Ecosystèmes Biodiversité, Evolution” («UMR 6553») Centre National de la Recherche Scientifique University Rennes 1 Rennes France; ^3^ SNSB—Zoologische Staatssammlung München Munich Germany

**Keywords:** barcoding, deadwood, experiment, host–parasitoid interaction, natural enemy, specialization

## Abstract

Most parasites and parasitoids are adapted to overcome defense mechanisms of their specific hosts and hence colonize a narrow range of host species. Accordingly, an increase in host functional or phylogenetic dissimilarity is expected to increase the species diversity of parasitoids. However, the local diversity of parasitoids may be driven by the accessibility and detectability of hosts, both increasing with increasing host abundance. Yet, the relative importance of these two mechanisms remains unclear. We parallelly reared communities of saproxylic beetle as potential hosts and associated parasitoid Hymenoptera from experimentally felled trees. The dissimilarity of beetle communities was inferred from distances in seven functional traits and from their evolutionary ancestry. We tested the effect of host abundance, species richness, functional, and phylogenetic dissimilarities on the abundance, species richness, and Shannon diversity of parasitoids. Our results showed an increase of abundance, species richness, and Shannon diversity of parasitoids with increasing beetle abundance. Additionally, abundance of parasitoids increased with increasing species richness of beetles. However, functional and phylogenetic dissimilarity showed no effect on the diversity of parasitoids. Our results suggest that the local diversity of parasitoids, of ephemeral and hidden resources like saproxylic beetles, is highest when resources are abundant and thereby detectable and accessible. Hence, in some cases, resources do not need to be diverse to promote parasitoid diversity.

## Graphical abstract

We investigated saproxylic beetles and parasitoid Hymenoptera reared from experimentally felled trees. We show that host diversity must not mandatory promote parasitoid diversity (pictures were used after the Creative Commons license (CC BY‐SA‐2.0 and CC BY‐3.0); copyright by U. Schmidt and M.A. Broussard).

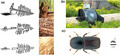

## 1 INTRODUCTION

Most species worldwide are natural enemies of other species, as exemplified by parasitoids and parasites. Those species tend to be neither specialized on a single host species nor completely generalist, colonizing a set of similar and often closely related hosts (Strong et al., 1984). Natural enemies have hence adapted to overcome the chemical, morphological, physiological, and immunological defense characteristics of their host species, which can be phylogenetically conserved (Gross, 1993; Strand & Pech, 1995). As a result, hosts with different functional characteristics or evolutionary ancestry tend to be exploited by different enemies (Frank, 1993). Hence, increasing phylogenetic or functional dissimilarity of host communities is thought to increase the species diversity of their enemies in a community (Schuler et al., 2015; Vialatte et al., 2010). This might be the case in systems in which functionally different hosts require fundamentally different adaptations from their enemies and the performance of an enemy to colonize one host species is traded off against its performance on colonizing another (Egas et al., 2005; Straub et al., 2011).

Enemy diversity may also depend on the detectability and accessibility of hosts: If hosts are hidden or sheltered, only the most specialized enemy species will be able to detect or use them (Price, 1973). Host detectability and accessibility, in turn, may increase with increasing host abundance. Host abundance increases host detectability by increasing olfactory, acoustical, or optical signals available for enemies (Yguel et al., 2014). Host abundance may also increase among‐host competition forcing to leave the enemy‐free space, that is, microsites or periods where or when hosts are inaccessible to enemies (Aukema & Raffa, 2000). Also, increased competition may require increased investment into competitiveness, binding energy that could otherwise be invested into defense against enemies (Bashey, 2015). In sum, low host abundance may reduce the diversity of natural enemies to a narrow subset of enemy species.

Parasitoids constitute several different taxonomic groups and are a key component of terrestrial ecosystems (Heraty, 2009). It is estimated that 10%–20% of all insect species belong to parasitoid Hymenoptera, representing the insect group with the highest species diversity worldwide (Forbes et al., 2018; Gaston, 1991). Comparable to parasites, the majority of parasitoid Hymenoptera is specialized on a certain set of hosts (Forbes et al., 2018), although many host species are so far unknown. As a result, a given parasitoid Hymenoptera species is typically most abundant only on one or a few focal host species.

Parasitoid Hymenoptera are one of the most important natural enemies of saproxylic beetle communities in forests, as mainly demonstrated for bark beetles (Curculionidae, Scolytinae) (Wegensteiner et al., 2015). Saproxylic beetles differ fundamentally in functional properties, such as in their preference for certain host tree species, diameters of deadwood, or their dependence on specific microclimatic conditions (Seibold et al., 2015). Moreover, communities of saproxylic beetles change with succession during deadwood decomposition (Parisi et al., 2018). Parasitoid Hymenoptera mainly attack the eggs, larvae, and pupae of saproxylic beetles and exhibit a clear preference for one developmental stage over the others (Strand & Pech, 1995; Wegensteiner et al., 2015). Many of these and other functional properties are phylogenetically conserved (Seibold et al., 2015). The functional or phylogenetic dissimilarity of saproxylic species might hence increase species diversity of parasitoid enemies. However, given that most saproxylic species are extremely ephemeral and hidden, the diversity of their parasitoids might also increase with saproxylic abundance.

We investigated communities of saproxylic beetle hosts and their associated parasitoid Hymenoptera. Both groups of insects were reared from experimentally felled trees during the early succession of deadwood. For each tree, the species diversity of saproxylic beetles and parasitoid Hymenoptera emerging from those beetles were quantified. In addition, the saproxylic beetle communities of each tree erized according to their similarities across seven functional traits and their phylogenetic similarity, to measure functional and phylogenetic dissimilarities. Our aim was to test whether abundance or functional and phylogenetic dissimilarity of host communities determines the abundance and species richness of associated parasitoid Hymenoptera.

## 2 MATERIALS AND METHODS

### 2.1 Study area and experimental design

Our study was conducted in the Bavarian Forest National Park, located in southeastern Germany. The park consists of approximately 24,850 ha of mountainous forests at elevations between 600 and 1,460 m a.s.l. Depending on the altitude, the annual average temperature ranges from 3.8 to 5.8°C. Yearly precipitation varies between 1,200 and 1,800 mm (Bässler et al., 2010). Forest stands in higher elevations of the park area are naturally dominated by Norway spruce (*Picea abies*). Within the last two decades, extensive waves of natural disturbances have generated highly diverse deadwood structures, contributing substantially to partially more than 300 m^3^ of deadwood per hectare (Thorn et al., 2017).

Observational studies do naturally depend on the occurrence and spatial distribution of natural disturbances, which typically do not match the requirements of a standardized scientific study design (Lindenmayer et al., 2010). Also, often only a single host species is dominant, and results might be idiosyncratic to that species. Hence, we created artificial windthrows and applied bark treatments to create variance in the community composition of saproxylic beetles, that is, to avoid the dominance of the European bark beetle (*Ips typographus*). *Ips typographus* is the most important pest species of mature spruce stands throughout Eurasia with a preference for weakened or freshly dead Norway spruce trees (Wermelinger, 2004).

**FIGURE 1:**
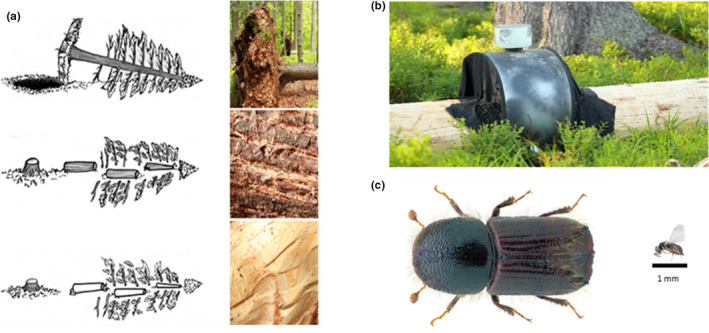
(a) Different mechanical bark treatments used to alter abundance and species composition of saproxylic beetle communities. On each plot, one tree was fully debarked, one tree was bark‐scratched (disruption of the phloem every 3 cm), and the third tree served as the control. (b) Stem emergence trap used for sampling of saproxylic beetles and parasitoid Hymenoptera. (c) The European spruce bark beetle (*Ips typographus*) and a parasitoid Hymenoptera (Superfamily Chalcidoidea) as representatives for the trapped species and analyzed communities (pictures were used after the Creative Commons license (CC BY‐SA‐2.0 and CC BY‐3.0); copyright by U. Schmidt and M.A. Broussard)

Our field experiment was established in April 2013. The design consisted of 12 artificial windthrows (plots), each composed of three pulled down mature Norway spruce trees with similar physical attributes (Figure 1). Two trees per plot were uprooted and debranched, and their root plates were cut off. One tree was bark‐scratched (disruption of the phloem every 3 cm), and a second tree was debarked (removal of all phloem). The remaining tree served as control. The minimum distance between plots was 200 m (for more details of the experimental design, see Thorn, Bässler, et al. (2016), Thorn, Bußler, et al. (2016). Within the conditions provided in our experiment, communities of beetles and parasitoids assembled naturally.

### 2.2 Arthropod sampling

Communities of saproxylic beetles and parasitoid Hymenoptera were sampled by using one stem emergence trap per tree, which were established from April to September 2014. Stem emergence traps are covering a given tree section and are only capturing individuals directly emerging from this section, representing the local community more complete the, for example, flight interception traps (Sverdrup‐Thygeson & Birkemoe, 2009). To preserve species material for further steps, we used 90% ethanol and emptied traps monthly. All trapped beetles were identified to the species level according to Freude et al. (1963–1984) and classified as saproxylic according to Schmidl & Bußler (2004). To identify species of parasitoid Hymenoptera, one leg of each specimen was removed and submitted to the Canadian Centre for DNA Barcoding for DNA sequence analysis. DNA was extracted according to Ivanova et al. (2006), and standardized primer sets were used to amplify and sequence the 658‐bp barcode region (Folmer et al., 1994; Hebert et al., 2003). The sequences were aligned with those in the Barcode of Life data system (Ratnasingham & Hebert, 2007). If the identification to the species level was not possible, barcoding data were used as molecular operational taxonomic units (MOTUs), which are represented by barcode index numbers and closely approximate species‐level identifications (Ratnasingham & Hebert, 2013). Hereafter, MOTUs are included in “species” for simplicity. Single MOTUs were defined by a 97% sequence similarity. Species or MOTUs with sequences that matched those of parasitoids certainly not using coleopteran hosts according to Yu et al. (2016) and Noyes (2019) were excluded. The beetle data used in our paper are available online in Chao et al. (2019).

### 2.3 Host phylogeny and traits

We used the phylogenetic tree of European saproxylic beetles provided by Seibold et al. (2015) according to the approach of Kuhn et al. (2011). The latter allows the input of partially resolved trees with known topology and node ages as constraints and applies a Markov chain Monte Carlo algorithm to permute polytomies using a constant‐rate birth–death model. The phylogenetic tree was calibrated using 25 calibration points obtained from fossil records.

Functional dissimilarity was analyzed using the resource‐related and morphological traits of saproxylic beetles described by Gossner et al. (2013), Thorn et al. (2014), and Seibold et al. (2015). These databases represent the most comprehensive information about saproxylic beetle functional traits in Central Europe. Information on the mean diameter niche position (<15, 15–35, 35–70, >70 cm), the decay niche position (alive, freshly dead, initiated, advanced decomposition, extremely decomposed), and the canopy cover niche (open, semi‐open, closed) was included, together with information on the preference of saproxylic beetle species for host trees (coniferous, broadleaved, both types of trees), the microhabitat guilds of larvae (wood and bark, cavities, fungi), the mean body size of single species, and the larval feeding type (detritivorous, mycetophagous, xylophagous, zoophagous). For the degree of phylogenetic correlation in functional traits, see Doerfler et al. (2020).

### 2.4 Dissimilarity of host communities

All statistical analyses were carried out in R version 4.0.3 (www.r‐project.org). The phylogenetic tree of European saproxylic beetles was used to quantify phylogenetic dissimilarity based on the mean nearest taxon distance (MNTD), reflecting the mean distance separating each species from its closest relative in the same community. MNTD was additionally used to quantify the similarities among co‐occurring species that may influence colonization by parasitoids. A value of MNTD less than zero thereby represents a clustered (i.e., similar) community of hosts, while a value greater than zero represents an overdispersed (i.e., dissimilar) community. To calculate MNTD, we first calculated the cophenetic distances between co‐occurring species using the function “cophenetic” from the R package “stats.” Because MNTD values can decrease as the number of species in a community increases, an abundance‐weighted null model was applied to compare the observed MNTD values with those of 999 randomly generated communities containing the same number of species by using the function “ses.mntd” from the R package “picante” (Cavender‐Bares et al., 2006; Kembel et al., 2010). This procedure resulted in a standardized effect size of MNTD that reflected the mean MNTD, that is, the phylogenetic dissimilarity of a beetle community standardized by species numbers. Standardized effect sizes of functional and phylogenetic diversities were only weakly correlated (Pearson's *r*: 0.29).

The functional dissimilarity of saproxylic beetle communities was calculated using the same procedure as described above, except that the distance matrices of traits were calculated as Euclidean (numeric variables) and Gower (non‐numeric or in combination with numeric variables) distances using the function “daisy” from the R package “cluster” (Gower, 1971).

### 2.5 Statistical analyses

The effects of abundance, species richness, phylogenetic dissimilarity, and functional dissimilarity of saproxylic beetle communities were tested on the abundance, species richness, and Shannon diversity of parasitoids as response variables. We standardized the number of sampled saproxylic beetle species to 0.95 sample completeness using a rarefaction/extrapolation framework according to Hsieh et al. (2016), implemented in the R package “iNext.” We estimated the sample coverage of saproxylic beetles and parasitoid Hymenoptera by the same approach.

The effect of saproxylic beetle communities on the abundance of parasitoids was tested by applying a generalized linear mixed model with poisson error distribution by using the R package “lme4” (Bates et al., 2015). This model included the abundance of parasitoids as response variable and the log‐transformed abundance, species richness, functional dissimilarity, and phylogenetic dissimilarity of the respective host beetle communities as predictor variables. Furthermore, we included the plot as random effect to account for the nested study design (Bolker et al., 2009) and the sample id to account for possible Poisson overdispersion (Elston et al., 2001). We used the same model formula to model species numbers and additionally included the log‐transformed abundance of parasitoid Hymenoptera to the model. Shannon diversity of parasitoids was modeled using the same model formula as for species numbers but a Gaussian error distribution.

## 3 RESULTS

**FIGURE 2:**
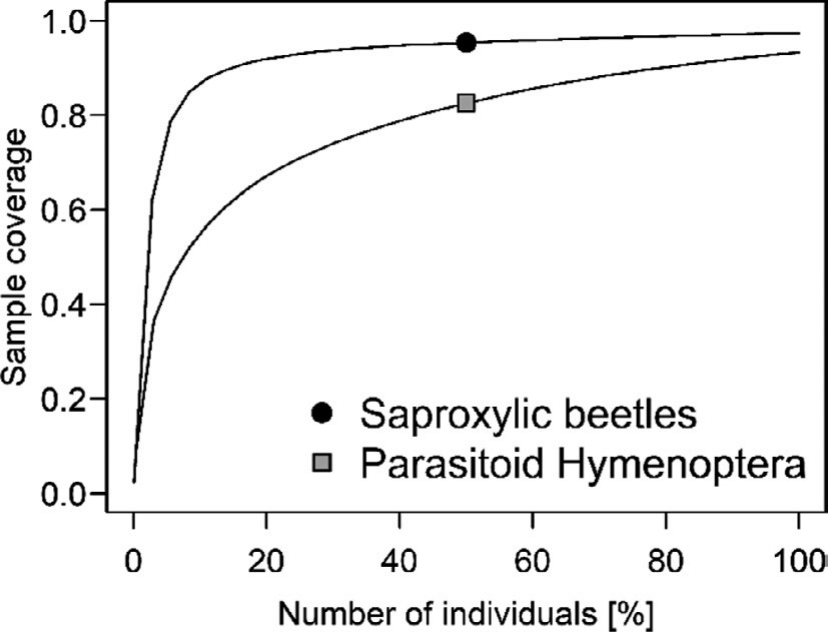
Sample coverage of saproxylic beetles and parasitoid Hymenoptera based on rarefaction/extrapolation up to twice the actual sampling effort. Note that the number of sampled individuals has been transformed to percentage for better comparability

Our dataset included 15,516 individuals of saproxylic beetles from 106 species and 146 individuals from 44 species of parasitoids (Table 1), corresponding to a 0.93% rate of successful parasitization. The mean species number per log was 23.61 ± 7.5 species for saproxylic beetles and 2.64 ± 2.6 for parasitoids. Species accumulation curves revealed a high sample coverage of both, saproxylic beetles and parasitoid Hymenoptera (Figure 2).

**TABLE 1:** Species of parasitoid Hymenoptera recorded in our study with their respective abundances

ID	Scientific name	Abundance
Braconidae (6 species/33 individuals)		
BOLD:AAU9839	*Chelonus* sp.	1
BOLD:ACM7216	*Ropalophorus clavicornis*	1
BOLD:ACM7563	*Dendrosoter middendorffii*	5
BOLD:ACQ8673	Brachistinae	1
BOLD:ACQ9535	*Cosmophorus regius*	2
BOLD:ACQ9771	*Blacus* sp.	23
Ceraphronidae (3 species/5 individuals)		
BOLD:ACF6025	*Aphanogmus* sp.	3
BOLD:ACG4508	*Aphanogmus* sp.	1
Ceraph_01	*Aphanogmus* sp.	1
Diapriidae (2 species/2 individuals)		
BOLD:ACG4055	*Pantoclis* sp.	1
Diapri_01	*Trichopria* sp.	1
Eulophidae (4 species/7 individuals)		
BOLD:ACQ8898	*Necremnus croton*	2
BOLD:ACQ9006	*Necremnus leucarthros*	3
Euloph_01		1
Euloph_02		1
Eupelmidae (1 species/2 individuals)		
Eupelm_01		2
Eurytomidae (1 species/3 individuals)		
BOLD:ACM7745	*Eurotoma arctica/afra*	3
Figitidae (1 species/1 individual)		
BOLD:ACQ9714	Eucoilinae	1
Ichneumonidae (4 species/13 individuals)		
BOLD:ABU6543	*Enclisis vindex*	2
BOLD:ACQ9146	*Phrudus monilicornis*	3
BOLD:ACR0681	*Phradis* sp.	1
BOLD:ACR0964	*Rhimphoctona teredo*	7
Platygastridae (10 species/36 individuals)		
BOLD:AAN8098	*Telenomus* sp.	9
BOLD:ACC2809	Scelioninae	2
BOLD:ACF7380		1
BOLD:ACF9487		2
BOLD:ACI4334	*Telenomus* sp.	10
BOLD:ACI4527	*Telenomus* sp.	1
BOLD:ACI9091	*Telenomus* sp.	4
BOLD:ACI9128	*Telenomus* sp.	3
BOLD:ACR1479		2
BOLD:ACR1888		2
Proctotrupidae (2 species/4 individuals)		
BOLD:ACR1488		2
BOLD:ACR1985		2
Pteromalidae (10 species/40 individuals)		
BOLD:AAN8215	*Pteromalus* sp.	2
BOLD:AAZ7417	*Mesopolobus gemellus*	3
BOLD:ACA9177		1
BOLD:ACM7334	*Roptrocerus mirus*	15
BOLD:ACM7652		2
BOLD:ACQ8466	*Dinotiscus eupterus*	3
BOLD:ACQ8826	*Holcaeus compressus*	7
BOLD:ACQ9876	*Perniphora robusta*	3
Pterom_01		3
Pterom_03		1

Saproxylic beetles were dominated by bark beetles (Curculionidae, Scolytinae), encompassing 10,869 individuals from 18 species. The most abundant beetle species, *Trypodendron lineatum*, was represented by 7,496 trapped individuals. *Ips typographus* was trapped by 824 (control trees), 180 (bark‐scratched trees), and 19 (debarked trees) individuals, respectively. Parasitoids were recorded from 11 families, dominated by Pteromalidae (40 individuals, 10 species), Platygastridae (36 individuals, 10 species), and Braconidae (33 individuals, 6 species). Many of the parasitoids recorded in our study were rare: 36 species of parasitoids with ≤3 individuals contributed 66 of the 146 sampled individuals (Table 1).

**FIGURE 3:**
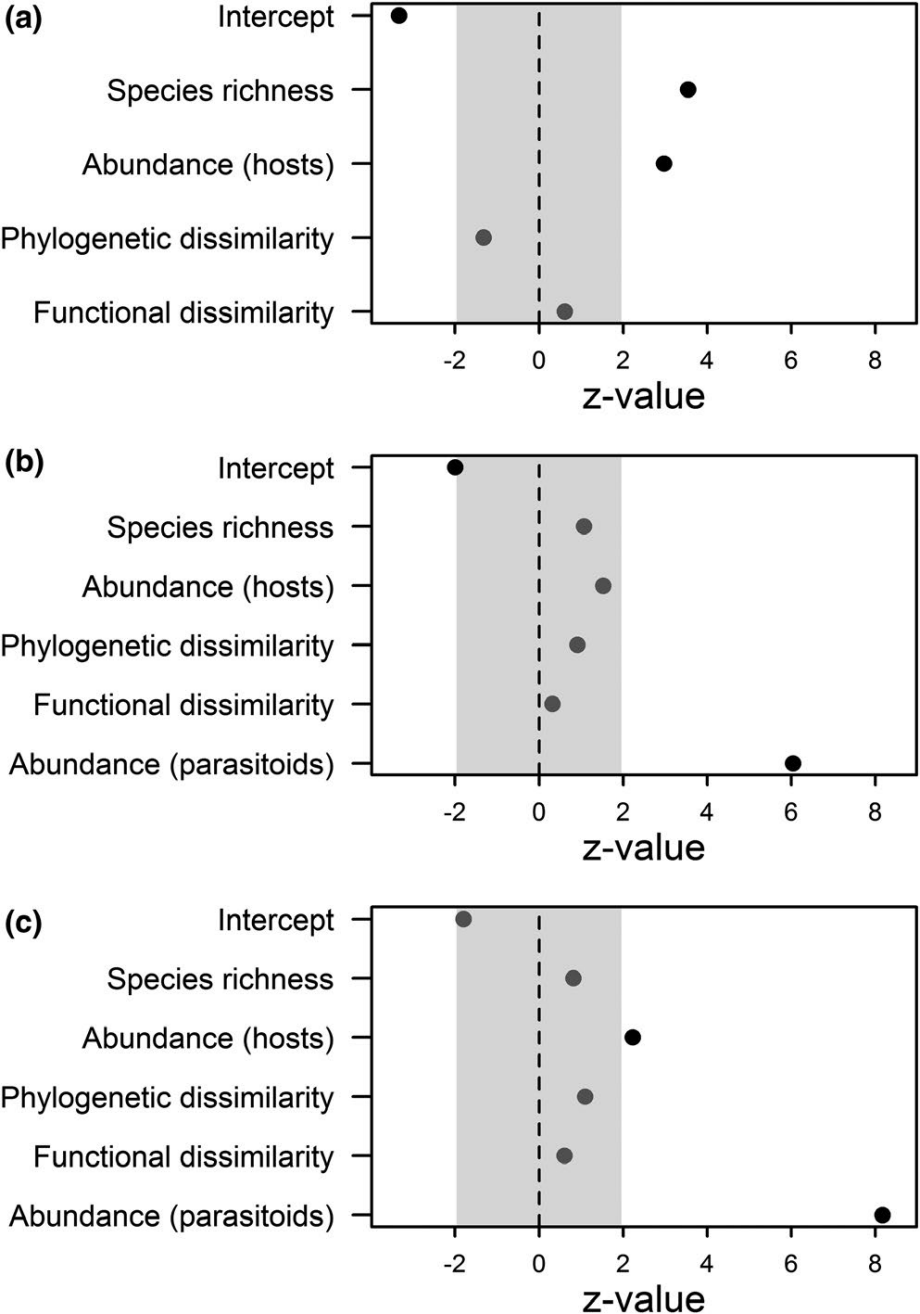
Relative effect strengths (*z*‐values) of predictors on (a) abundance of parasitoid Hymenoptera, (b) species numbers of parasitoid Hymenoptera, and (c) Shannon diversity of parasitoid Hymenoptera. Gray shading indicates the range of nonsignificant *z*‐values, significant effects depicted by black dots

Abundance of parasitoids increased with increasing abundance and species richness of saproxylic beetles (Figure 3a). Species numbers and Shannon diversity of parasitoid Hymenoptera strongly increased with increasing abundance of parasitoid Hymenoptera (Figure 3b,c). Shannon diversity of parasitoid Hymenoptera increased with increasing host abundance (Figure 3c). We did not find any significant effect of functional or phylogenetic dissimilarity on any of our response variables (Figure 3).

## 4 DISCUSSION

Our results indicate that abundance and species richness in communities of parasitoid Hymenoptera increase with increasing abundance of hosts, whereas functional and phylogenetic dissimilarities of host communities were of minor importance. Furthermore, species numbers of parasitoids were strongly promoted by their abundance, which in turn increases with species richness of hosts. Thereby, our results do not support that functional or phylogenetic dissimilarity of hosts begets the diversity of parasitoids within deadwood objects, but rather point toward an abundance‐driven system.

The increasing abundance and species richness of parasitoids with increasing abundance of their saproxylic beetle hosts are in line with the »more individuals hypothesis«, an extension of the »species‐energy theory« (Clarke & Gaston, 2006; Schuler et al., 2015). The »more individuals hypothesis« predicts an increase in the abundance and consequently in species richness of a given species group (i.e., parasitoids) in response to an increase in the availability of resources (i.e., hosts). The »more individuals hypothesis« is valid as long as species use different niches and do not rely on the same resources (MacArthur & MacArthur, 1961; Tews et al., 2004). In our study system, an increasing abundance could increase the detectability (Vinson, 1998) and accessibility (Price, 1973) of saproxylic beetle hosts.

In our study system, host accessibility may be limited, given the short spatiotemporal window during which a specific developmental stage of a host exists. Furthermore, parasitoids may be limited to a specific set of host species and do not utilize patches with extremely low host abundance (Hassell, 2000; Murdoch, 1977).

High host abundances may force some hosts into enemy‐exposed microenvironments. In our study system, this could apply to bark beetles, which reached highest abundances of potential hosts and then might be forced to occupy parts of the tree trunk which are more easier to access to parasitoids (Aukema & Raffa, 2000).

Higher abundances of hosts might also increase the detectability, that is the olfactory signal to parasitoids, since saproxylic beetles, especially bark beetles, emit sex pheromones (Vega & Hofstetter, 2015). Furthermore, at extremely high abundances, bark beetles can emit anti‐aggregation pheromones (Sun et al., 2006). Such pheromones might additionally increase host detectability at high abundances. Overall, abundance of bark beetles might render them distinctly more detectable or accessible to a larger number of parasitoid species and thereby increase their species diversity.

We did not find any effect of functional or phylogenetic dissimilarity of hosts on parasitoid communities (Figure 3). Functional dissimilarity might to some degree reflect functional identity. If there is one dominant functional group, such as in our case bark beetles, then a greater functional similarity may reflect the dominance of that group. Specifically, our samples were composed of species colonizing mainly the phloem of recently killed Norway spruce. Specialization on particular host groups may reflect constraints on, for example, ovipositor length. For example, bark thickness limits parasitization by *Caenopachys hartigii* (Braconidae, Doryctinae) (Mancini et al., 2003). Thus, host species that do not feed directly under the bark or other superficial parts of deadwood might not be reachable by this enemy. A highly abundant bark beetle species in our study was *T. lineatum*, which create galleries into sapwood, where it might be difficult to access for parasitoids. Indeed, only few species of parasitoids enter bark beetle galleries for oviposition (Vega & Hofstetter, 2015).

It is possible that the effect of functional identity overlays and partly conceals the effect of functional dissimilarity, explaining why we did not find any effect of functional dissimilarity. Despite we used the most comprehensive database of saproxylic beetles, we are still lacking information about, for example, temporal occurrence of saproxylic beetles. An extension of our trait database by such information may hence change the relative importance of functional dissimilarity. Phylogenetic dissimilarity did not show a significant effect on parasitoid abundance (Figure 3). One of the reasons for the lacking effect of phylogenetic dissimilarity might be that closely related beetle hosts occur in high abundances, such as bark beetles in our study system. This is ultimately caused by the fact that host beetle communities cannot be established in a fully experimental design, that is, covering a range of phylogenetic dissimilarity, due to their host tree specializations. Moreover, for a set of locally coexisting species, phylogenetic dissimilarity may not necessarily reflect the dissimilarity of functional traits (Prinzing et al., 2008).

Overall, our study suggests that resource diversity does not mandatory begets the diversity of local enemy communities. This intuitive hypothesis implicitly assumes that any given type of resources is detectable and accessible to the consumers. In that case, a diverse set of resources can be used by a diverse set of consumers. However, if resources are hosts that are ephemeral and hidden, they may easily remain undetected by or inaccessible to many potential parasitoids. Only at high host abundance, detection and access may become possible for many parasitoid species, leading to an increase of parasitoid diversity with host abundance.

## CONFLICT OF INTEREST

The authors declare no conflict of interest.

## AUTHOR CONTRIBUTIONS

**Sebastian Vogel:** Conceptualization (equal); Formal analysis (equal); Investigation (equal); Writing‐original draft (lead); Writing‐review & editing (equal). **Andreas Prinzing:** Conceptualization (equal); Writing‐review & editing (equal). **Heinz Bußler:** Data curation (equal); Resources (equal). **Jörg Müller:** Resources (equal); Writing‐review & editing (equal). **Stefan Schmidt:** Data curation (equal); Investigation (equal); Resources (equal). **Simon Thorn:** Conceptualization (equal); Formal analysis (equal); Project administration (equal); Resources (equal); Supervision (equal); Writing‐original draft (equal); Writing‐review & editing (equal).

## Data Availability

All specimen data are accessible in the Barcode of Life Database (BOLD, http://www.boldsystems.org) as a single citable dataset (https://dx.doi.org/10.5883/DS‐GBBWA). The data include collecting locality, geographic coordinates, elevation, collector, one or more digital images, identifier, and voucher depository.
